# Acetate rescues defective brain-adipose metabolic network in obese Wistar rats by modulation of peroxisome proliferator-activated receptor-γ

**DOI:** 10.1038/s41598-021-98605-5

**Published:** 2021-09-23

**Authors:** Kehinde Samuel Olaniyi, Morounkeji Nicole Owolabi, Chukwubueze Lucky Atuma, Toluwani Bosede Agunbiade, Bolanle Yemisi Alabi

**Affiliations:** 1 Cardio/Repro-Metabolic and Microbiome Research Unit, Department of Physiology, College of Medicine and Health Sciences, Afe Babalola University, P.M.B. 5454, Ado-Ekiti, 360101 Nigeria; 2 Department of Medical Microbiology and Parasitology, College of Medicine and Health Sciences, Afe Babalola University, Ado-Ekiti, 360101 Nigeria; 3 Department of Hematology and Virology, University of Medical Science Teaching Hospital Complex, Akure, Nigeria

**Keywords:** Physiology, Endocrinology

## Abstract

We investigated the hypothesis that acetate ameliorates brain-adipose metabolic dysfunction (BAMED) in high fat diet (HFD)-induced obesity, possibly by modulation of peroxisome proliferator-activated receptor-γ (PPAR-γ). Ten-week-old male Wistar rats were randomly assigned into four groups (n = 6/group): Control, acetate and obese with or without acetate groups received vehicle (distilled water; *po*), acetate (200 mg/kg, *po*) and 40% HFD with or without acetate respectively. The treatments lasted for 12 weeks. Obese animals showed increase in body weight, visceral fat mass, insulin and triglyceride-glucose index and a reduction in insulin sensitivity. In addition, obese animals also showed increase in plasma/hypothalamic and adipose pyruvate dehydrogenase kinase-4, lactate-pyruvate ratio, malondialdehyde, γ-glutamyl transferase, and a decrease in glucose-6-phosphate dehydrogenase, glutathione, nitric oxide and PPAR-γ. HFD also elevated plasma/hypothalamic lipid and decreased adipose lipid profile, increased hypothalamic and adipose tumor necrosis factor-α, interleukin-6 and histone deacetylase (HDAC), and elevated plasma/adipose leptin. These alterations were reversed by concomitant administration of acetate. The present results demonstrate that obesity is characterized by BAMED, which is accompanied by altered HDAC/PPAR-γ. The results in addition suggest that acetate, an HDAC inhibitor rescues BAMED with consequent normalization of body weight and visceral fat mass by modulation of PPAR-γ and suppression of oxidative stress.

## Introduction

Obesity is a complex multifactorial metabolic disease, affecting more than 30% of global population as overweight or obese, and attributing to 8% of the annual worldwide mortality^1^. Based on the current prevalence, over 38% of the global population is estimated to be overweight or obese by 2030^2,3^. Obesity was initially evident to be a problem of the developed countries and even as it continues to rise in many of these countries, its prevalence continues to trend upward in developing countries, including among the children and adolescents^2,4^. It is usually characterized with excessive adiposity and any of the following: insulin resistance, hyperglycemia and overweight^1,5^. Obesity constitutes a risk factor for a number of chronic diseases including type 2 diabetes mellitus (T2DM), cardiovascular diseases (CVDs), depression, renal disease, liver disease, reproductive dysfunction and cancer, and has adverse effects on overall health as well as global public health^5–7^. In fact, the economic and psychosocial burden of obesity coupled with its comorbidities is rapidly escalating.

The cause of obesity has been linked to behavioral (including sedentary lifestyle and consumption of high calorie-rich nutrients), environmental and genetic factors^8,9^. Previous studies reported that genetics influence the body mass index (BMI) which is a commonly used indicator of obesity and accounts for energy requirements, fuel utilization and muscle metabolic characteristics^10–12^, however, the current obesity epidemic has been shown to be principally driven by behavioral and environmental factors^1^. Sedentary lifestyle and consumption of high energy diet are the most common contributors to the energy imbalance that causes overweight and excessive fat deposition^7,9^. Nevertheless, the pathogenic processes underlying obesity are multifactorial and still under elucidation which posit a barrier for designing effective strategies towards prevention and management.

However, a number of previous investigations have documented leptin resistance as a critical factor in the development of obesity^13^. Physiologically, leptin is secreted by adipocytes in proportion to total fat mass^14^ and regulates energy balance by reducing energy intake or increasing energy expenditure^13^. It reduces energy intake by decreasing food or caloric intake through appetite inhibition in the hypothalamus, thereby preventing excess body weight^15^. It also increases energy expenditure by elevating lipid-glucose utilization, thus limiting excess energy storage or excessive fat deposition in the adipose tissue^13,16^. The regulatory functions of leptin occur by binding to hypothalamic leptin receptors, which signals to downstream effectors through the Janus kinase/signal transducers and activators of transcription factors (JAK/STAT) signaling pathway^13^. On the other hand, impaired leptin tolerance elicits metabolic signals that promote energy storage inform of fat deposition and caloric intake, which are crucial features of obesity, thus associating obesity with high leptin concentration^15^. Earlier studies have demonstrated leptin resistance in experimental rodents fed with high fat diet (HFD)^17^, suggesting that HFD consumption limits the action of leptin in the regulation of energy balance regardless of its concentration. Similarly, hyperleptinemia could also induce or aggravate leptin insensitivity by upregulating the suppressor of cytokine signalling-3 (SOCS-3) with consequent blockade of signal transduction of leptin^17,18^.

In addition, the development of obesity which is characterized by leptin resistance has been associated with inflammation both in peripheral tissues including adipose tissue and in hypothalamic areas critical for energy homeostasis^19–21^. Study by Thaler et al., reported the evidence of hypothalamic inflammatory signaling in rodents within 1 to 3 days of HFD exposure, prior to substantial weight gain^22^, suggesting the possible involvement of hypothalamic inflammation in the pathogenesis of obesity, unlike inflammation in the peripheral tissues which develops as a consequence of obesity. This study added that both reactive gliosis and markers suggestive of neuron injury were evident in the hypothalamic arcuate nucleus of rodents within the first week of HFD exposure and extends to mediobasal hypothalamus with chronic exposure^22^. Nevertheless, evidence reveals hypothalamic lesion in obese humans as assessed by magnetic resonance imaging^23^, suggesting that obesity is critically associated with hypothalamic injury in both humans and experimental rodents. Similarly, more investigations have demonstrated elevated level of circulating pro-inflammatory cytokines, proteases and growth factors derived from adipose tissue of obese rodents, which possibly contributes to an increased hypothalamic inflammatory signaling that mediates leptin resistance and weight gain^17,20,21^. However, the cellular interactions underlying the brain-adipose inflammatory response in the development of obesity is not clear.

Peroxisome proliferator-activated receptor-γ (PPAR- γ) is among the three isoforms of PPARS, which are nuclear hormone receptors that exert transcriptional control on genes housing PPAR-responsive regulatory elements and heterodimerize with retinoid X receptors^24,25^. PPARS are activated or repressed in response to regulatory networks of genes controlling various physiological processes that involve inflammation, adipogenesis, lipid-glucose metabolism and insulin sensitivity^26,27^. Defects in PPARs have been linked to metabolic stress and related syndrome, including the impaired adipose tissue remodeling and function^28^, which are detrimental during nutrient deprivation when adipose tissue remodeling is required for cellular survival. Earlier studies have demonstrated PPARs as lipid sensors that are capable of improving energy homeostasis when optimally activated^29^. Nonetheless, considering the therapeutic potential of PPARS activation in metabolic stress and related disorders, it would be of clinical relevance and public health interest to investigate the possible involvement of PPARs, especially PPAR-γ in brain-adipose inflammatory events underlying obesity.

Epigenetic modulation of key metabolic enzymes contributes to the pathogenesis of obesity^30,31^. Post-translational modification of proteins involving acetylation or deacetylation are governed by histone acetyltransferases (HAT) and histone deacetylases (HDACs) which transfers acetyl group to histone proteins and causes reversal of acetylation respectively. Evidence exists that the PPAR-γ gene is not only genetically regulated, but also regulated by epigenetic modulation, including histone modification and its regulatory enzymes such as HAT and HDACs^32,33^. Similarly, the functional roles of epigenetic enzymes, including HDACs in the PPAR-γ transcriptional pathway have been suggested^34,35^. In addition, certain post-translational modifications could also be mediated by pro-inflammatory and pro-oxidant species leading to suppression of gene expression^36^. Therefore, therapies targeting HDACs might be a potential promoter of PPAR-γ with possible attenuation of visceral adiposity, and in addition be a novel safer and effective intervention for the management of obesity. Acetate is a short chain fatty acid (SCFA) metabolite produced from distal gut microbiota that has been attributed to a wide range of protective health benefits^37^. Acetate is known to regulate metabolic activities of cells by inhibiting HDAC and/or activating G-protein coupled receptors signaling. It improves intestinal mucosa integrity/immunity, enhance insulin sensitivity and exert anti-inflammatory effects. Earlier studies demonstrated that oral supplementation with acetate exerts recovery from hepatic lipotoxicity^38,39^, neuroinflammation^40^, and hypertension^41^. However, the effects of acetate on disrupted brain-adipose metabolic link and/or brain-adipose inflammatory events underlying obesity was not known. The present study was therefore designed to test the hypothesis that SCFAs, acetate ameliorates defective brain-adipose metabolic network associated with high fat diet (HFD)-induced obesity in male Wistar rats, possibly by modulation of PPAR-γ.

## Results

### Acetate attenuates excess body weight gain and visceral fat mass in high fat diet-induced obese male rats

**Figure 1:**
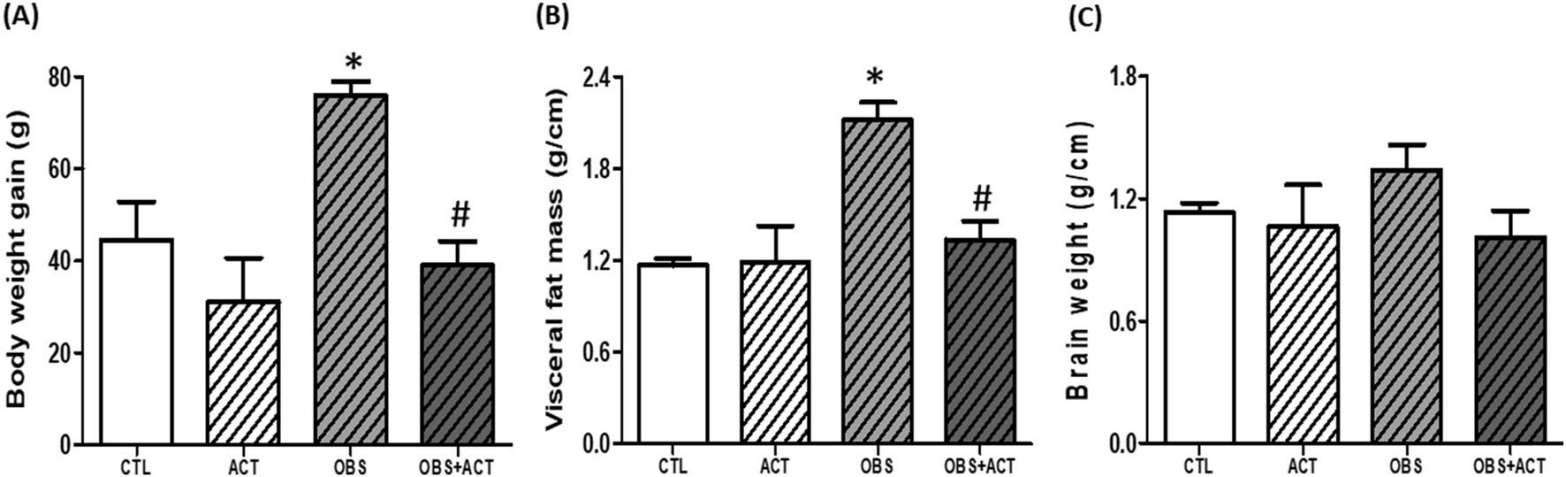
Effects of acetate on body weight gain (**A**), visceral fat mass (**B**) and brain weight (**C**) in high fat diet-induced obese Wistar rats. Body weight gain and visceral fat mass but not brain weight increased in obese rats, which were attenuated following the administration of acetate. Data are expressed as mean ± SD. n = 6 and analyzed by one-way ANOVA followed by Bonferroni post hoc* test.* (*p < 0.05 vs. CTL; ^#^*p* < 0.05 vs. OBS). *CTL* Control; *OBS* Obesity, *ACT* Acetate.

There was a significant increase (p < 0.05) in body weight and visceral fat mass in obese animals compared with control animals. However, the administration of acetate to obese plus acetate (OBS + ACT) group significantly reduced (p < 0.05) the body weight and visceral fat mass compared to the untreated obese animals and no significant difference when compared to the control group (Fig. 1A,B). Although, the brain weight did not significantly change in all the experimental groups compared to the control group (Fig. 1C).

### Acetate restores glucose homeostasis in high fat diet-induced obese male rats

**Figure 2:**
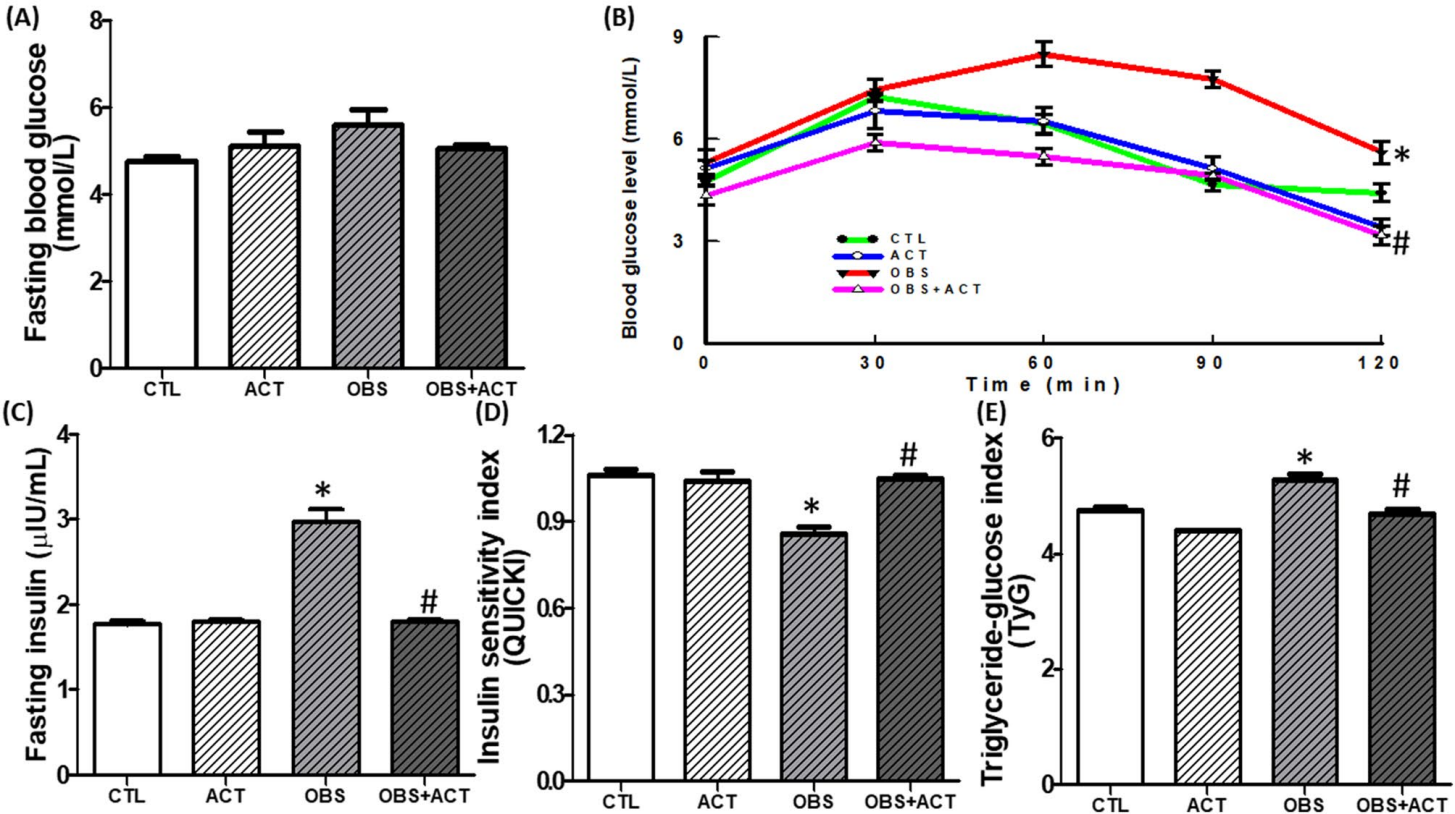
Effects of acetate on fasting blood glucose (**A**), oral glucose tolerance (**B**), fasting insulin (**C**), insulin sensitivity (**D**) and triglyceride-glucose index (**E**) in high fat diet-induced obese Wistar rats. The fasting blood glucose did not change, glucose tolerance was impaired, fasting insulin increased, insulin sensitivity decreased and TyG (surrogate marker of insulin resistance) increased in obese rats, which were attenuated following the administration of acetate. Data are expressed as mean ± SD. n = 6 and analyzed by one-way ANOVA, except for the oral glucose tolerance, where data were analyzed by repeated measures ANOVA followed by Bonferroni post hoc* test.* (*p < 0.05 vs. CTL; ^#^*p* < 0.05 vs. OBS). *CTL* Control, *OBS* Obesity, *ACT* Acetate, *QUICKI* Quantitative check for insulin sensitivity index, *TyG* triglyceride-glucose index.

Though the fasting blood glucose remained unchanged (p < 0.05) but a significant decrease in glucose tolerance and insulin sensitivity were observed in obese animals compared with control animals and these were significantly increased (p < 0.05) following the administration of acetate to OBS + ACT group compared to the untreated obese group and no significant difference when compared to the control group. In addition, there was a significant increase (p < 0.05) in fasting plasma insulin and triglyceride-glucose (TyG) ratio in obese animals compared with control animals and these were normalized following the administration of acetate to OBS + ACT group compared to the untreated obese group (Fig. 2).

### Acetate improves glucoregulatory enzymes in high fat diet-induced obese male rats

**Figure 3:**
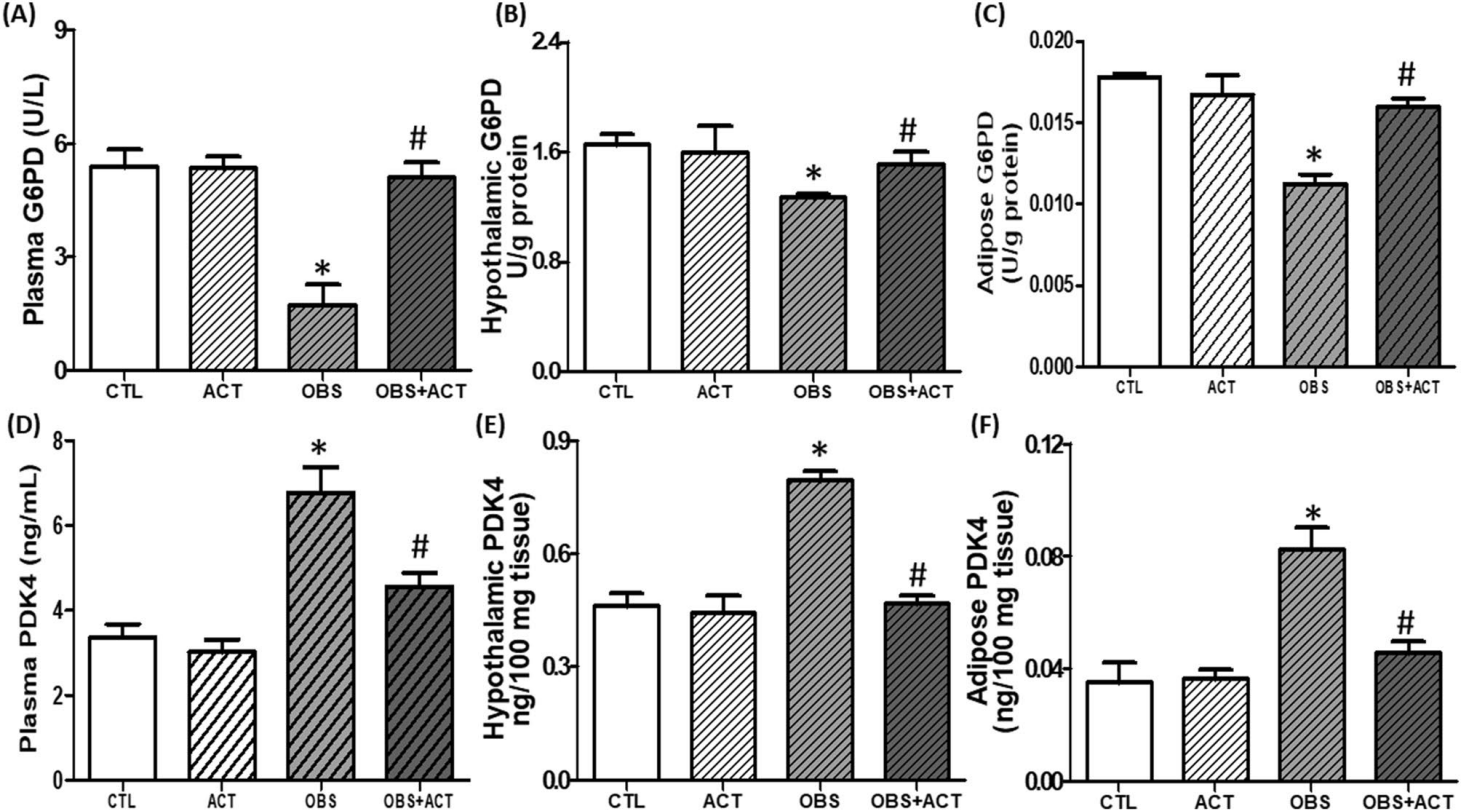
Effects of acetate on plasma, hypothalamic and adipose Glucose-6-phosphate dehydrogenase (**A**–**C**) and Pyruvate dehydrogenase kinase-4 (**D**–**F**) in high fat diet-induced obese Wistar rats. Plasma, hypothalamic and adipose G6PD decreased while PDK4 increased in obese rats, which were attenuated following the administration of acetate. Data are expressed as mean ± SD. n = 6 and analyzed by one-way ANOVA followed by Bonferroni post hoc test. (*p < 0.05 vs. CTL; ^#^*p* < 0.05 vs. OBS). *CTL* Control, *OBS* Obesity, *ACT* Acetate, *G6PD* Glucose-6-phosphate dehydrogenase, *PDK-4* Pyruvate dehydrogenase kinase-4.

There was a significant decrease (p < 0.05) in plasma, hypothalamic and adipose glucose 6 phosphate dehydrogenase (G6PD) with corresponding increase in pyruvate dehydrogenase kinase-4 (PDK4) in obese animals compared with control animals. However, administration of acetate significantly increased (p < 0.05) the plasma, hypothalamic and adipose G6PD with corresponding decrease in PDK4 in OBS + ACT group compared to the untreated obese group and no significant difference when compared to the control group (Fig. 3).

### Acetate decreases lactate-pyruvate ratio and γ-glutamyl transferase (GGT) activity in high fat diet-induced obese male rats

**Figure 4:**
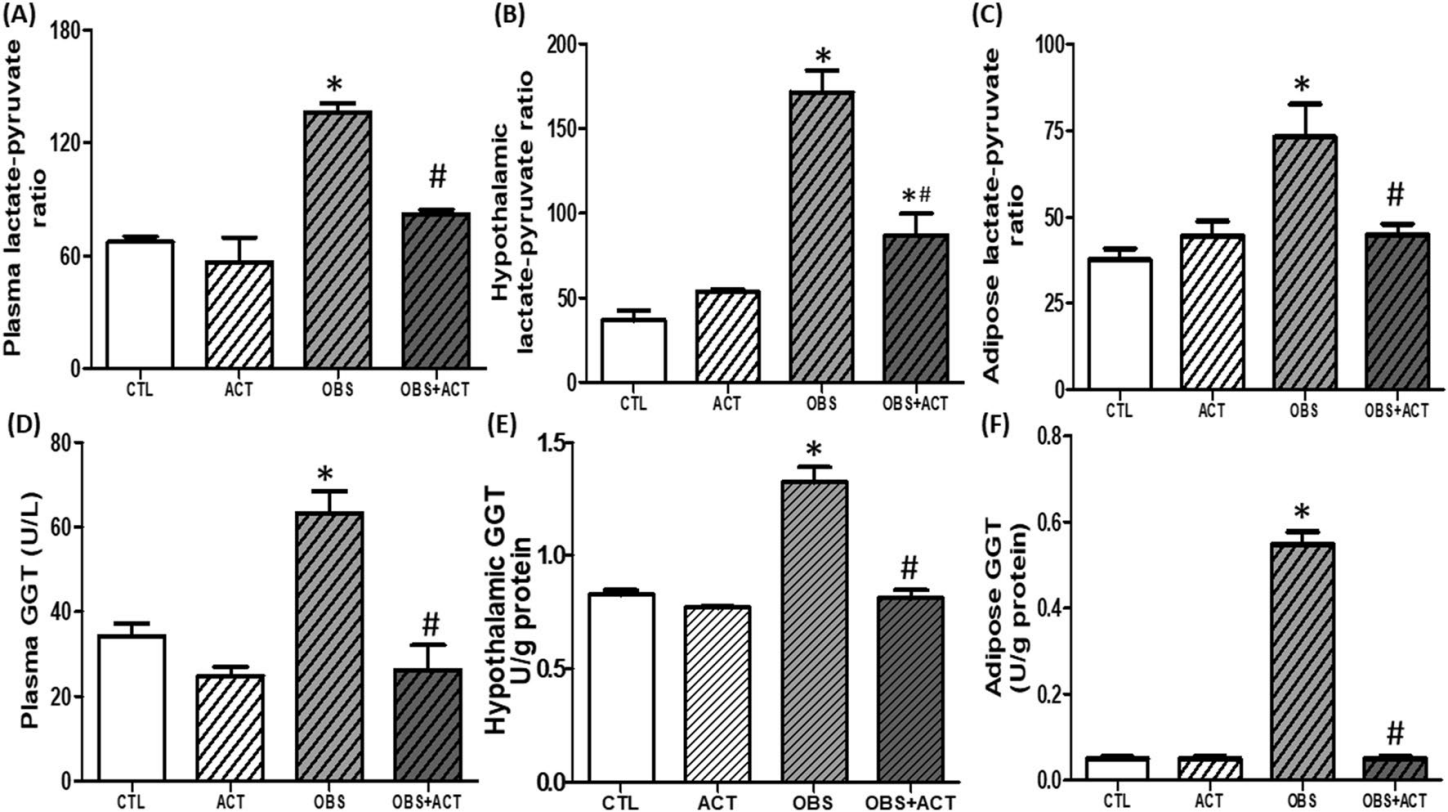
Effects of acetate on plasma, hypothalamic and adipose lactate-pyruvate ratio (**A**–**C**) and γ-Glutamyl transferase activity (**D**–**F**) in high fat diet-induced obese Wistar rats. Plasma, hypothalamic and adipose lactate-pyruvate ratio and GGT increased in obese rats, which were attenuated following the administration of acetate. Data are expressed as mean ± SD. n = 6 and analyzed by one-way ANOVA followed by Bonferroni post hoc* test.* (*p < 0.05 vs. CTL; ^#^*p* < 0.05 vs. OBS). *CTL* Control, *OBS* Obesity, *ACT* Acetate, *GGT* γ-Glutamyl transferase.

There was a significant increase (p < 0.05) in plasma, hypothalamic and adipose lactate-pyruvate ratio with corresponding increase in GGT activity in obese animals compared with control group. Nevertheless, administration of acetate significantly decreased (p < 0.05) the plasma, hypothalamic and adipose lactate-pyruvate ratio and GGT activity in OBS + ACT group compared to the untreated obese animals and no significant difference when compared to the control group. Nonetheless, hypothalamic lactate-pyruvate ratio was significantly higher OBS + ACT group when compared to the control group (Fig. 4).

### Acetate normalizes lipid profile in high fat diet-induced obese male rats

**Figure 5:**
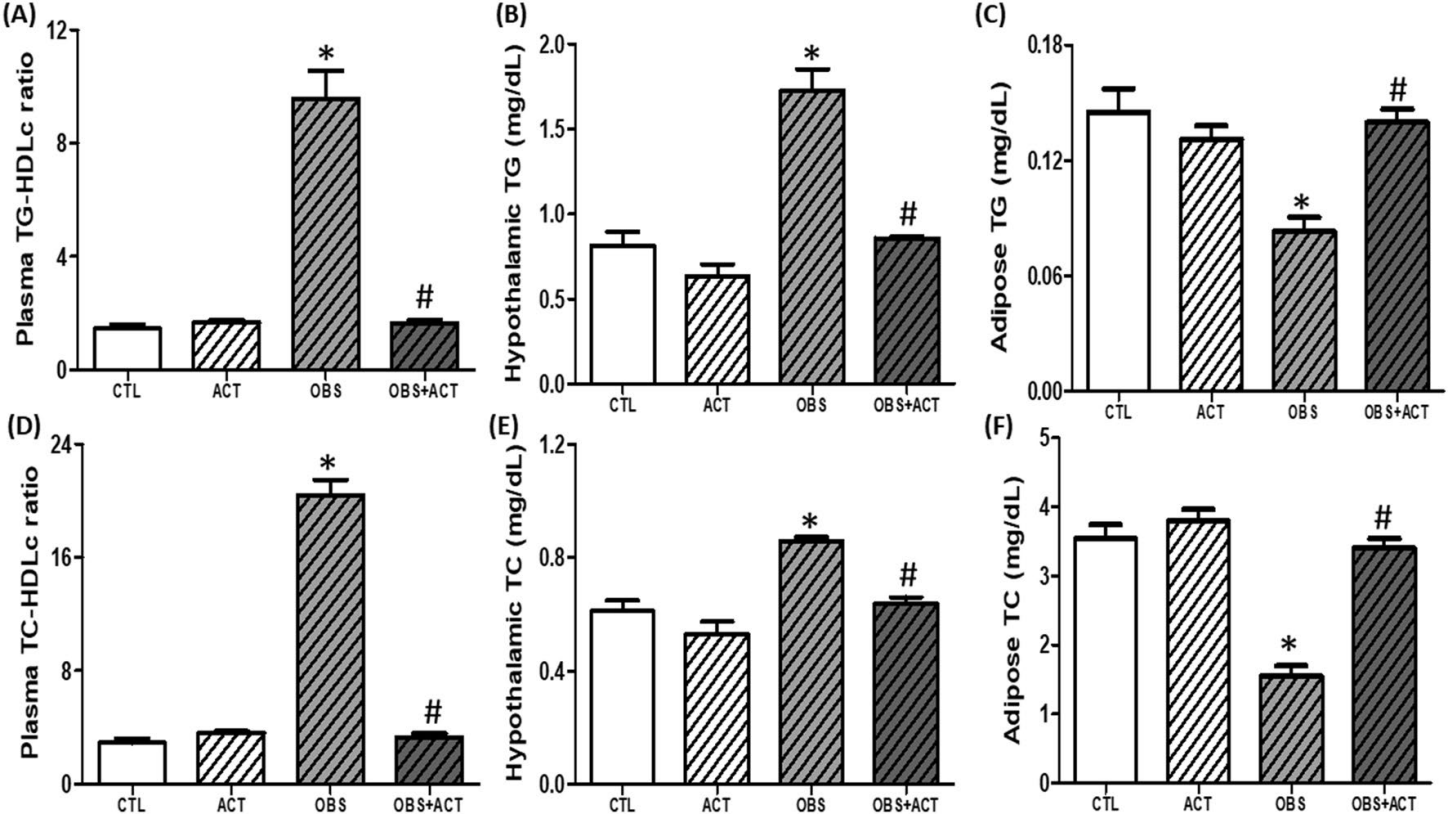
Effects of acetate on plasma, hypothalamic and adipose lipid profile (**A**–**F**) in high fat diet-induced obese Wistar rats. Plasma TG/HDLc and TC/HDLc and hypothalamic TG and TC increased while adipose TG and TC decreased in obese rats, which were reversed following the administration of acetate. Data are expressed as mean ± SD. n = 6 and analyzed by one-way ANOVA followed by Bonferroni post hoc test. (*p < 0.05 vs. CTL; ^#^*p* < 0.05 vs. OBS). *CTL* Control, *OBS* Obesity, *ACT* Acetate, *TG* Triglyceride, *TC* total cholesterol, *HDLc* High-density lipoprotein cholesterol.

There was a significant increase (p < 0.05) in plasma triglyceride/high-density lipoprotein cholesterol (TG/HDLc) and total cholesterol/HDLc (TC/HDLc) and hypothalamic TG and TC in obese animals compared with control animals. However, administration of acetate attenuated dyslipidemia and hypothalamic lipid accumulation in OBS + ACT group compared to the untreated obese group. Besides, adipose TG and TC decreased significantly (p < 0.05) in obese animals compared with control animals, which were attenuated following the administration of acetate in OBS + ACT group compared to the untreated obese group and no significant difference when compared to the control group (Fig. 5).

### Acetate attenuates lipid peroxidation and glutathione depletion in high fat diet-induced obese male rats

**Figure 6:**
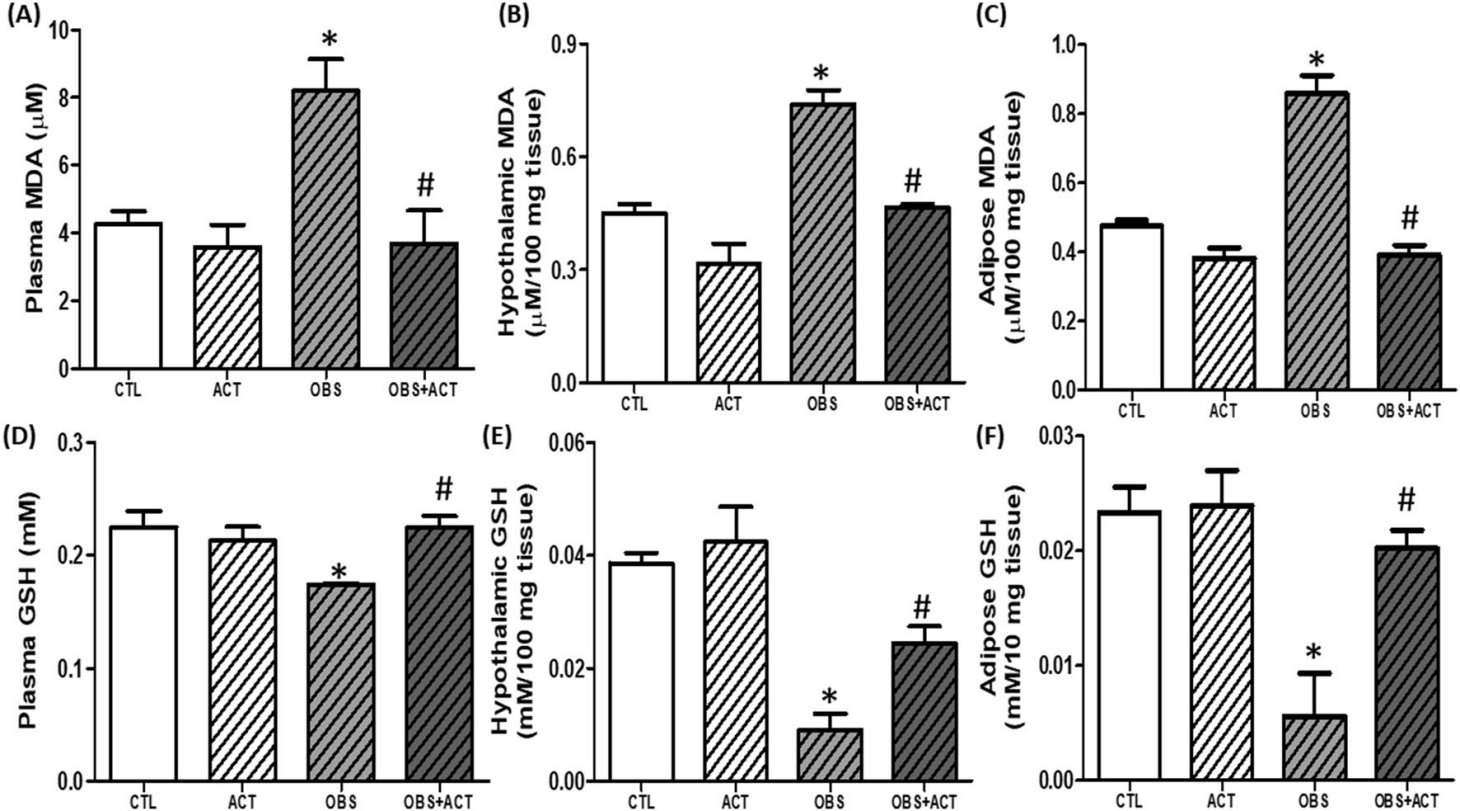
Effects of acetate on plasma, hypothalamic and adipose malondialdehyde (**A**–**C**) and glutathione (**D**–**F**) in high fat diet-induced obese Wistar rats. Plasma, hypothalamic and adipose MDA increased while glutathione decreased in obese rats, which were attenuated following the administration of acetate. Data are expressed as mean ± SD. n = 6 and analyzed by one-way ANOVA followed by Bonferroni post hoc* test.* (*p < 0.05 vs. CTL; ^#^*p* < 0.05 vs. OBS). *CTL* Control, *OBS* Obesity, *ACT* Acetate, *MDA* Malondialdehyde, *GSH* Glutathione.

There was a significant increase (p < 0.05) in plasma, hypothalamic and adipose malondialdehyde (MDA) and a decrease in glutathione (GSH) concentration in obese animals compared with control animals. Nevertheless, administration of acetate significantly decreased plasma, hypothalamic and adipose MDA with corresponding increase in GSH concentration in OBS + ACT group compared to the untreated obese group and no significant difference when compared to the control group (Fig. 6).

### Acetate decreases hypothalamic and adipose inflammatory biomarkers in high fat diet-induced obese male rats

**Figure 7:**
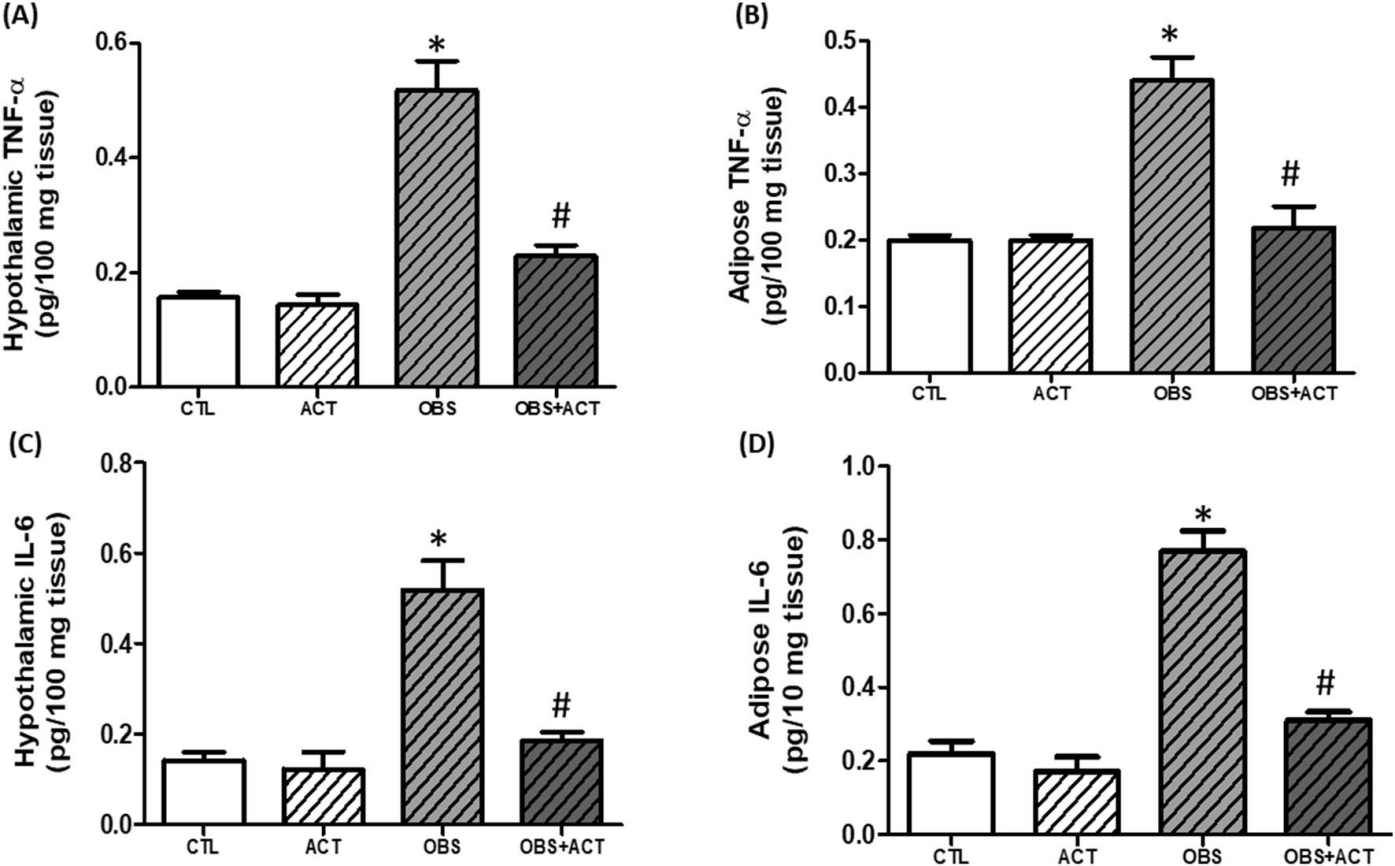
Effects of acetate on hypothalamic and adipose TNF-α (**A**–**C**) and IL-6 (**D**–**F**) in high fat diet-induced obese Wistar rats. Hypothalamic and adipose TNF-α and IL-6 increased in obese rats, which were attenuated following the administration of acetate. Data are expressed as mean ± SD. n = 6 and analyzed by one-way ANOVA followed by Bonferroni post hoc* test.* (*p < 0.05 vs. CTL; ^#^*p* < 0.05 vs. OBS). *CTL* Control, *OBS* Obesity, *ACT* Acetate, *TNF-α* Tissue necrosis factor-α, *IL-6* Interleukin-6.

There was a significant increase (p < 0.05) in hypothalamic and adipose tumor necrosis factor-α (TNF-α) and interleukin-6 (IL-6) in obese animals compared with control animals and these alterations were reversed following the administration of acetate to OBS + ACT group compared to the untreated obese group and no significant difference when compared to the control group (Fig. 7).

### Acetate increases circulating, hypothalamic and adipose nitric oxide concentration in high fat diet-induced obese male rats

**Figure 8:**
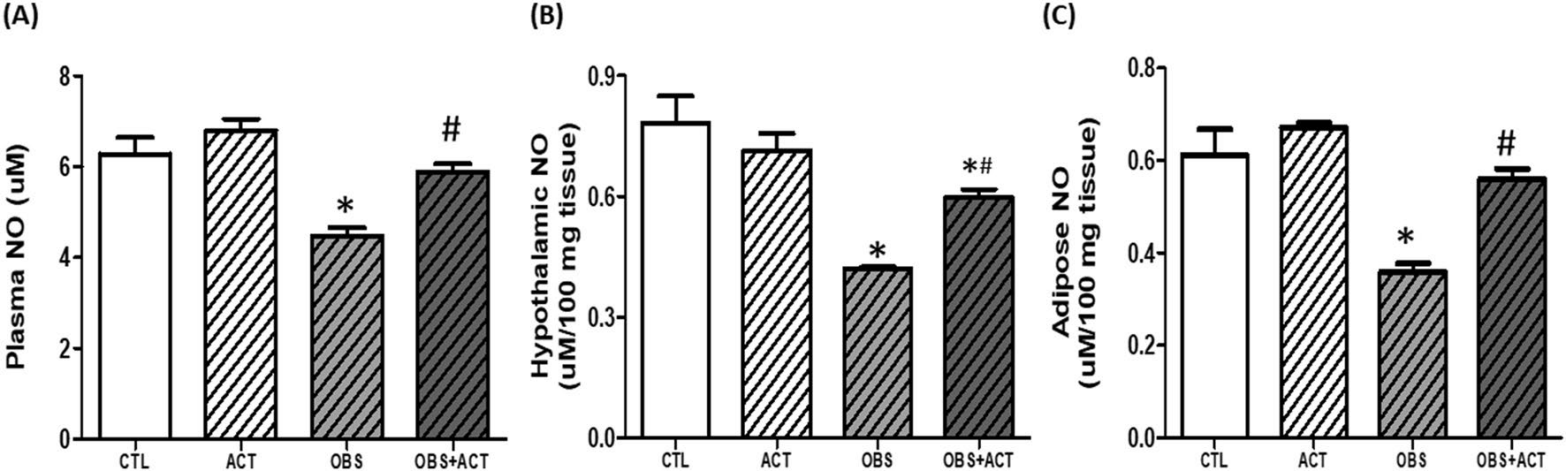
Effects of acetate on plasma, hypothalamic and adipose nitric oxide concentration (**A**–**C**) in high fat diet-induced obese Wistar rats. Plasma, hypothalamic and adipose NO increased in obese rats, which were reduced following the administration of acetate. Data are expressed as mean ± SD. n = 6 and analyzed by one-way ANOVA followed by Bonferroni post hoc* test.* (*p < 0.05 vs. CTL; ^#^*p* < 0.05 vs. OBS). *CTL* Control, *OBS* Obesity, *ACT* Acetate, *NO* Nitric oxide.

There was a significant decrease (p < 0.05) in plasma, hypothalamic and adipose nitric oxide concentration in obese animals compared with control animals. However, administration of acetate significantly increased (p < 0.05) the plasma, hypothalamic and adipose nitric oxide (NO) concentration in OBS + ACT group compared to the untreated obese group and no significant difference when compared to the control group (Fig. 8).

### Acetate decreases circulating and adipose leptin concentration in high fat diet-induced obese male rats

**Figure 9:**
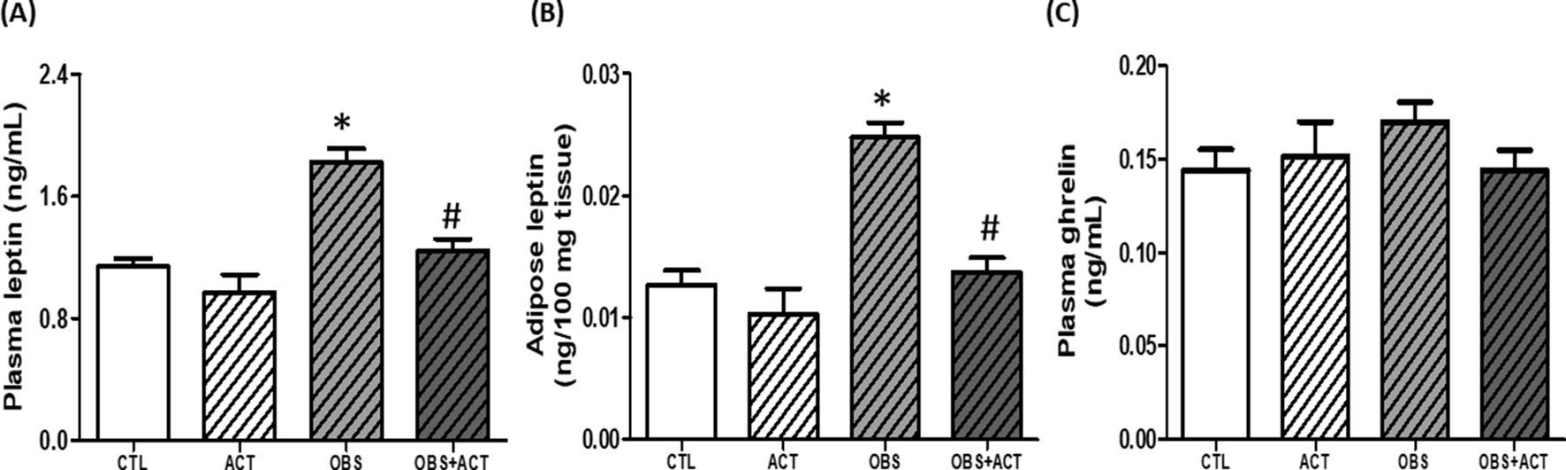
Effects of acetate on plasma and adipose leptin (**A**,**B**) and plasma ghrelin concentration in high fat diet-induced obese Wistar rats. Plasma and adipose leptin increased while ghrelin concentration remained unchanged and the alteration in leptin concentration was attenuated by the administration of acetate. Data are expressed as mean ± SD. n = 6 and analyzed by one-way ANOVA followed by Bonferroni post hoc* test.* (*p < 0.05 vs. CTL; ^#^*p* < 0.05 vs. OBS). *CTL* Control, *OBS* Obesity, *ACT* Acetate.

There was a significant increase (p < 0.05) in plasma and adipose leptin concentration in obese animals compared with control animals. However, administration of acetate normalized the circulating and adipose leptin concentration in OBS + ACT group compared to the untreated obese group. Nevertheless, plasma ghrelin concentration was not altered in obese group compared to the control group (Fig. 9).

### Acetate decreases hypothalamic and adipose HDAC in high fat diet-induced obese male rats

**Figure 10:**
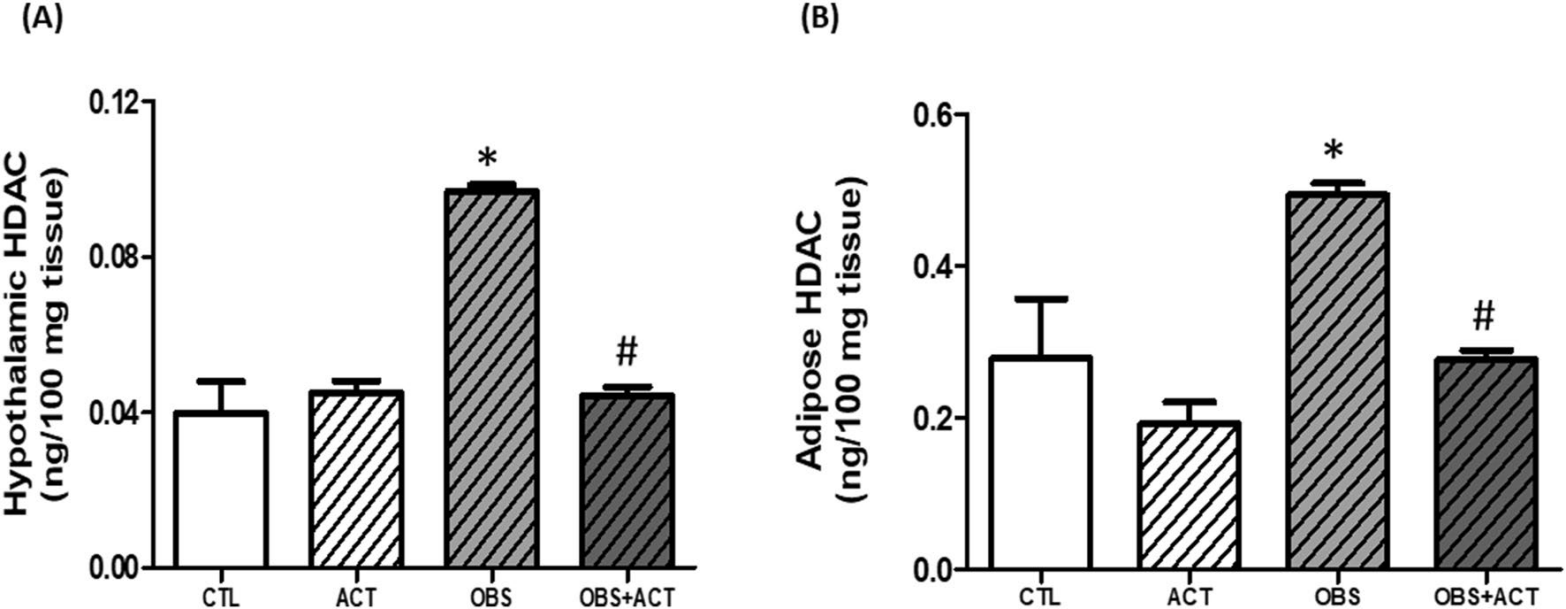
Effects of acetate on hypothalamic and adipose histone deacetylase (**A**,**B**) in high fat diet- induced obese Wistar rats. Hypothalamic and adipose HDAC, which were restored by the administration of acetate. Data are expressed as mean ± SD. n = 6 and analyzed by one-way ANOVA followed by Bonferroni post hoc* test.* (*p < 0.05 vs. CTL; ^#^*p* < 0.05 vs. OBS). *CTL* Control, *OBS* Obesity, *ACT* Acetate, *HDAC* Histone deacetylase.

The hypothalamic and adipose levels of HDAC significantly increased (p < 0.05) in obese animals compared with control animals. Nevertheless, administration of acetate significantly decreased (p < 0.05) the hypothalamic and adipose HDAC levels in OBS + ACT group compared to the untreated obese group and no significant difference when compared to the control group (Fig. 10).

### Acetate increases circulating, hypothalamic and adipose PPAR-γ in high fat diet-induced obese male rats

**Figure 11:**
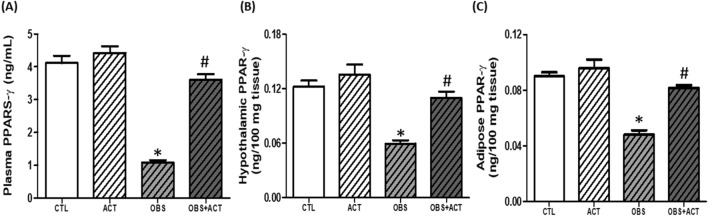
Effects of acetate on plasma, hypothalamic and adipose Peroxisome proliferator-activated receptor-γ (**A**–**C**) in high fat diet-induced obese Wistar rats. Plasma, hypothalamic and adipose PPAR-γ decreased in obese rats, which were elevated by the administration of acetate. Data are expressed as mean ± SD. n = 6 and analyzed by one-way ANOVA followed by Bonferroni post hoc* test.* (*p < 0.05 vs. CTL; ^#^*p* < 0.05 vs. OBS). *CTL* Control, *OBS* Obesity, *ACT* Acetate, *PPAR-γ* Peroxisome proliferator-activated receptor-γ.

The plasma, hypothalamic and adipose levels of PPAR-γ significantly increased (p < 0.05) in obese animals compared with control animals. Nonetheless, administration of acetate significantly increased (p < 0.05) the plasma, hypothalamic and adipose levels of PPAR-γ in OBS + ACT group compared to the untreated obese group and no significant difference when compared to the control group (Fig. 11).

## Discussion

**Figure 12:**
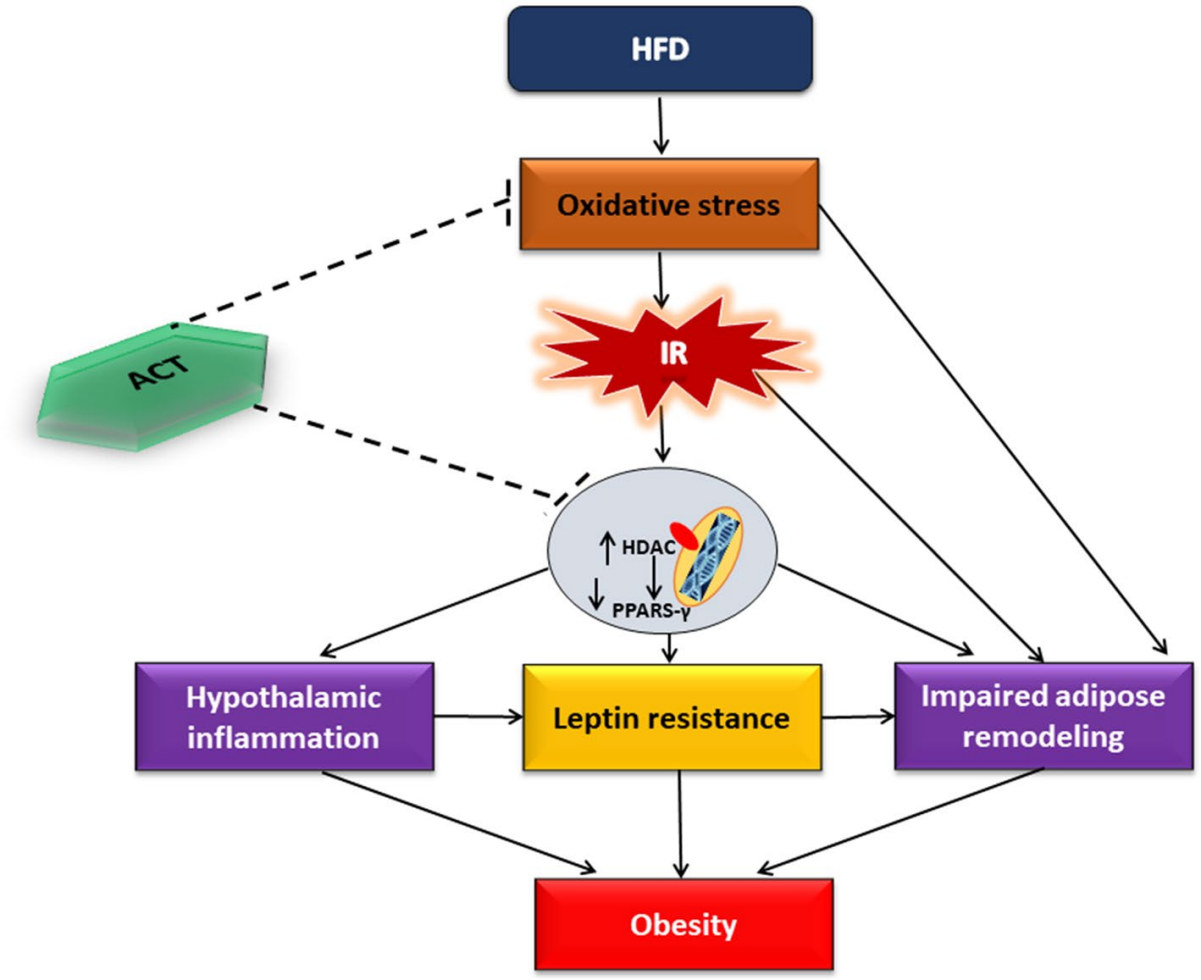
Schematic diagram showing the possible effects of acetate and the involvement of PPAR-γ in brain-adipose network in the development of obesity. *HFD* High fat diet, *ACT* Acetate, *PPAR-γ* Peroxisome proliferator-activated receptor-γ, *HDAC* Histone deacetylase.

The key finding from the present study is that acetate as HDAC inhibitor attenuates defective hypothalamic-adipose metabolic network in HFD-induced obesity and this beneficial effect of acetate is accompanied by elevated PPAR-γ and suppressed oxidative stress. The results of the study in general showed that HFD induces obesity, which is characterized with increased body weight, visceral fat mass, fasting insulin and triglyceride-glucose index and decreased insulin sensitivity with unaltered blood glucose when compared to the control group. In addition, obese animals also showed increased plasma, hypothalamic and adipose PDK4, lactate-pyruvate ratio, GGT, lipid peroxidation (MDA) and decreased antioxidant capacity (G6PD and GSH), NO and PPAR-γ, elevated plasma and hypothalamic lipid and decreased adipose lipid profile, increased hypothalamic and adipose inflammation (TNF-α, IL-6) and HDAC with corresponding increase in adipose and circulating leptin compared to the control group. However, administration of acetate reversed these alterations with consequent normalization of body weight and visceral fat mass by suppression of oxidative stress and upregulation of PPAR-γ (Fig. 12).

Obesity has been well characterized by excess body weight and visceral fat mass with or without insulin resistance in both experimental rodents and humans^5,7,9^. The present results that high fat diet induced obesity with evidence of increased body weight gain and visceral adiposity when compared to the control group show consistency with earlier observations. As extension, the results showed that the present model of obesity is associated with insulin resistance and hyperinsulinemia as revealed with elevated TyG/reduced insulin sensitivity and increased fasting insulin concentration respectively. Although the fasting blood glucose was not affected when compared to control group despite the impaired glucose tolerance observed in obese animals. The findings here suggest that in addition to excess body weight or visceral adiposity, the obese animals are characterized with disrupted glucose homeostasis regardless of the normal blood glucose level that might have resulted from the compensatory hyperinsulinemia. Besides, the findings also imply that the obese animals are pre-diabetic, which is a dysmetabolic state of glucose level that is primarily characterized by impaired glucose tolerance, thus validating earlier observations that obesity constitutes an independent risk factor to T2DM and attendant CVDs^5–7^. Nevertheless, the brain weight of obese animals was not affected when compared to the control group, although the development of obesity has been associated with changes in organ weight, particularly, the liver, kidney and heart^42,43^. However, other studies have reported that changes in organ weight is dependent on the etiology of obesity^44,45^.

Disruption of glucose homeostasis observed in obese animals is a reflection of impaired glucose metabolism^46,47^ which is corroborated by dysregulated glucoregulatory enzymes, especially G6PD and PDK4, an isoform of PDK. Glucose 6 phosphate dehydrogenase and PDK4 are critical metabolic enzymes that are involved in energy production inform of ATP either through glycolysis or pentose phosphate pathway (PPP)^48,49^. Glucose 6 phosphate dehydrogenase is the first step and rate-limiting enzyme in the oxidative stage of PPP, which results in production of ribose-5-phosphate^49^, while PDK, including PDK4 inhibits pyruvate dehydrogenase, an enzyme that regulates the oxidative metabolism of glucose through pyruvate dehydrogenase complex that promotes the conversion of pyruvate to acetyl coA^50,51^. In the present study, there was a significant decrease in plasma, hypothalamic and adipose G6PD with corresponding increase in PDK4 in obese animals compared to control group. These results therefore indicate that glucose metabolism or utilization is impaired in obese animals, which seems similar to previous studies, including studies from our laboratory that reported elevated PDK in animal models of obesity^52,53^ and in obese humans^54^. In addition, impaired glucose metabolism or utilization for ATP production favors conversion of pyruvate into lactate as revealed by elevated plasma, hypothalamic and adipose lactate-pyruvate ratio in obese animals compared with control group. Lactate-pyruvate ratio is a marker of cytosolic redox status^55^, and its increased level in obese animals possibly triggered hypothalamic and adipose tissue ischemia which moved both tissues toward proinflammatory phenotypes (elevated TNF-α and IL-6) and oxidative stress (increased MDA and decreased GSH) and aggravated by NO depletion. These perhaps led to tissue injuries as biochemically characterized by elevated circulating, hypothalamic and adipose GGT activity in obese animals compared to the control group. Therefore, the present findings suggest that impaired glucose metabolism or utilization at least in part promotes hypothalamic-adipose tissue injuries in obese animals.

Furthermore, the development of insulin resistance in obese animal reduced sensitivity of metabolic tissues including adipose tissue to insulin action as revealed by QUICKI, this possibly increased lipolysis with corresponding increase in circulating lipids such as TG/HDLc and TC/HDLc resulting in excess lipid influx into the non-metabolic tissues, including hypothalamic tissue with correspondent elevated TG and TC compared to the control group. The deposition of excess lipid in the hypothalamus has been earlier reported to reduce insulin sensitivity with corresponding decrease in hypothalamic insulin-mediated glucose uptake^56^. This possibly caused hypothalamic metabolic stress or lipotoxicity that triggered hypothalamic inflammatory response as revealed by elevated levels of hypothalamic TNF-α and IL-6 in obese animals compared to the control group. The observation here is in consonance with previous studies that reported the evidence of hypothalamic inflammatory signaling in rodents within 1 to 3 days of HFD exposure, prior to substantial weight gain due to energy imbalance^22^. Besides, hypothalamic metabolic stress could also be responsible for increased lipid peroxidation (MDA) with corresponding decrease in GSH, which possibly triggered hypothalamic oxidative stress with consequent injuries as revealed by elevated GGT activity in obese animals compared to the control group. On the other hand, adipose TG and TC significantly decreased in obese animals compared to control group. This observation is consistent with earlier studies, including a recent study from our laboratory, which reported that insulin-resistant adipose tissue is characterized with increased white visceral adiposity, that remodels and expands causing adipose dysfunction with corresponding decrease in adipose lipids including TG and FFA^57,58^ due to increased lipolysis. This further aggravates systemic and peripheral insulin resistance that underlie complications associated with obesity^5–7^. In addition, previous studies reported that HFD provoked insulin resistance-driven adipose remodeling, which often overwhelmed in chronic condition causing numerous macrophages invasion surrounding the dead adipocytes, thus triggering a crosstalk between the lipid, macrophages and toll like receptors located on the macrophages with consequent induction of proinflammatory cytokines^59,60^ as validated in the present study with elevated levels of circulating and adipose TNF-α and IL-6 in obese animals compared to the control group. The elevated levels of TNF-α and IL-6 in the adipose tissue are due to the enlargement of white adipose tissue (increased adiposity), which characterized obese animals causing the recruitment and activation of immune cell subsets in the white adipose tissue, which systemically induces the low-grade inflammation that in turns aggravate systemic and peripheral insulin resistance, contributing to the complications of obesity as earlier reported^25,60^. Therefore, the results herein imply that hypothalamic-adipose-lipid dysmetabolism drives hypothalamic/adipose inflammatory response that characterized obesity.

Besides, increase in visceral adipocyte possibly contributed to elevated leptin production in obese animals compared with control group. This observation is in consonance with earlier studies that reported a positive correlation between leptin and adipocytes in obese humans^14,61,62^. However, hypothalamic inflammatory response reduced the sensitivity of the hypothalamus to leptin (leptin resistance) leading to an increase in the plasma concentration of leptin in obese animals compared to control group. Hence the present results are consistent with earlier investigations by Thaler and Schwartz and Bittencourt et al., who demonstrated hyperleptinemia with consequent energy dysregulation in obese rodents that suffer hypothalamic lesions predominant with inflammation^20,21^. In addition, the results of the present study did not show significant alteration in the circulating level of ghrelin in obese animals compared to the control group, though earlier studies have shown inconsistent results in the status of ghrelin in obese individuals^63–65^. Therefore, the findings from the present data suggest that obesity may be independent of ghrelin concentration but is characterized by hyperleptinemia, which is attributable to increased visceral adiposity and leptin resistance invoked by hypothalamic inflammatory mediators.

Notably, there was a significant reduction in the plasma, hypothalamic and adipose concentration of PPARS-γ in obese animals compare to the control group. This reduction in PPAR-γ corresponded with increased visceral adiposity and bodyweight as well as impaired hypothalamic and adipose gluco-lipid metabolism, excessive lipid peroxidation, G6PD/GSH and NO depletion, elevated TNF-α/IL-6 and plasma or adipose leptin concentration that characterized obese animals. Similar to the present observations, deficiency of PPARs has been demonstrated in metabolic stress with consequent impaired adipogenic remodeling^28^, a pathological process that also occurs during adipose expansion and often results in nitric oxide deficiency and elevated inflammatory mediators as observed in obese animals compared to control group. Besides, earlier studies have demonstrated that administration of PPAR-γ ligands such as thiazolidinediones exert insulin sensitizing and anti-inflammatory effects through action on adipocytes and are thus widely used to treat metabolic-related disorders such as T2DM and atherosclerosis among others^25^. The present results in addition showed a significant increase in hypothalamic and adipose HDAC in obese animals when compared to the control group, which seems to be in agreement with earlier studies that identified many epigenetic regulators, including HDAC as an interacting partner with PPAR-γ^35,66^. Similarly, the unliganded PPAR-γ heterodimeric complex is reported to be associated with a multicomponent corepressor complex that contains HDAC activity. After binding to the PPAR-γ ligand, the corepressor complex is dismissed, and the coactivator complex that possesses histone acetylase activity is recruited to the PPAR-γ heterodimer. This leads to chromatin remodeling, which facilitates active transcription^67^. Therefore, HDAC inhibitor could be a potential agonist of PPAR-γ and a possible suitable therapeutic intervention to obesity.

Intriguingly, administration of acetate, an inhibitor of HDAC significantly increased plasma, hypothalamic and adipose PPAR-γ concentration with corresponding improvement in insulin sensitivity and glucose regulation as well as hypothalamic and adipose lipid metabolism, lipid peroxidation, G6PD/GSH-dependent antioxidant capacity, NO, inflammatory mediators and normalized leptin concentration, visceral adiposity and body weight in OBS + ACT group compared to untreated obese group. A number of studies have documented acetate as HDAC inhibitor^39,68,69^, and acetate has been earlier shown to improve insulin sensitivity and exert anti-inflammatory effects against neuroinflammation, hypertension and hyperandrogenism among others^38,40,41,70^. Similarly, the present results are also in line with the observation of Lee et al., who revealed that the inhibition of HDAC by MPT0E014 attenuated cardiomyopathy by upregulation of PPAR-γ^71^. However, to the best of our knowledge, the results of the present study suggest for the first time that inhibition of HDAC by acetate ameliorates disrupted hypothalamic-adipose metabolic network with consequent normalization of visceral adiposity in HFD-induced obesity by modulation of PPAR-γ. Although the present study is not without a limitation in such that the molecular mechanism underlying the regulatory role of acetate was not fully investigated. Nevertheless, the present data provides justification for future investigation of molecular mechanism and possibly holds public health interest or clinical relevance for prevention and management of obesity and its comorbidities.

In conclusion, the present results demonstrate that obesity is characterized with aberrant brain-adipose metabolic network, which is accompanied by altered HDAC and PPAR-γ. The results in addition suggest that acetate, an HDAC inhibitor rescues defective brain-adipose metabolic network with consequent normalization of body weight/visceral fat mass and metabolic homeostasis by modulation of PPAR-γ and suppression of oxidative stress. Hence, nutritional modifications that incorporate acetate engendered high fiber diet might be beneficial to obese individuals.

## Materials and methods

### Experimental animals and grouping

The study was conducted in accordance with the National Institutes of Health Guide for the Care and Use of Laboratory Animals and the protocol was approved by the Ethical Review Board of Afe Babalola University, Ado-Ekiti, Nigeria (ABUADERC/2020/06), and every effort was made to minimize both the number of animals used and their suffering. All the experiments were also performed and reported in accordance with the ARRIVE guidelines 2.0. Ten-week-old male Wistar rats weighing 160–180 g were procured from the animal house of the College of Health Sciences, Afe Babalola University, Ado-Ekiti. Rats had unhindered access to standard rat chow and tap water. After two weeks of acclimatization, the animals were randomly assigned into four groups (n = 6 per group) namely: Control (CTL), ACT-treated group, OBS group and OBS + ACT group. Rats were maintained in a colony under standard environmental conditions of temperature (22–26 °C), relative humidity (50–60%), and 12-h dark/light cycle.

### Induction of obesity

Obesity was experimentally induced by exposing the animals to 40% high fat diet prepared as described previously^72^ and administered for 12 weeks.

### Treatment

Control group received vehicle (standard rat chow and distilled water; *po*), ACT-treated group received standard rat chow and sodium acetate (200 mg/kg; *po*)*,* OBS group receive 40% HFD and distilled water (*po*0 and OBS + ACT-treated group received 40% high fat diet plus 200 mg/kg of sodium acetate. The treatment lasted for 12 weeks. Initial and final body weights were determined as shown in the supplementary table 1, and weight gain was estimated.

### Determination of visceral fat mass

After dissection, visceral fat mass was assessed as previously described^58,73^ by isolating and weighing the total perirenal, retroperitoneal, and abdominal fat pad and corrected to tibial length to eliminate variability.

### Oral glucose tolerance test (OGTT) and insulin resistance (IR)

The OGTT was performed 48 h prior to the sacrifice of the rats. The rats had 12-h overnight fast. Animals were loaded with glucose (2 g/kg; *po*). Blood was obtained from the tail before glucose load and then sequentially at 30, 60, 90 and 120 min. Glycemic levels were determined with a hand-held glucometer (ONETOUCH-LIFESCAN, Inc., Milpitas). Insulin sensitivity was determined using quantitative insulin sensitivity check index (QUICKI), which is expressed as 1/[log (fasting insulin) + log (fasting glucose)^73,74^, while IR was determined using its surrogate, triglyceride-glucose index, which is expressed as TyG = Ln (TG (mg/dl) × FPG (mg/dl)/2)^51,53,75^.

### Sample preparation

At the end of the treatment, the rats were anesthetized with sodium pentobarbital (50 mg/kg; *iv*). Blood was collected by cardiac puncture into heparinized tube and was centrifuged at 704 g for 5 min at room temperature. Plasma was stored frozen until it is required for biochemical assay.

### Preparation of brain and adipose tissue homogenates

After weighing the brain and visceral fat mass, 100 mg section of the hypothalamus and visceral fat tissue were carefully removed and homogenized with a glass homogenizer in phosphate buffer solution, centrifuged at 8000 *g* for 10 min at 4 °C.

### Biochemical analysis

#### Insulin concentration

The plasma level of insulin was determined using Rat ELISA kits with Catalog No. IS130D obtained from Calbiotech Inc. (Cordell Ct., El Cajon, CA 92,020, USA). This method is based on the direct sandwich technique in which two monoclonal antibodies are directed against separate antigenic determinants on the insulin molecule.

#### Leptin and ghrelin

The plasma and tissue concentration of leptin was determined using Rat ELISA kits with Catalog No. E-EL-R0582 obtained from Elabscience Biotechnology Inc. (Wuhan, Hubei, P.R.C., China). Similarly, plasma concentration of ghrelin was determined using Rat ELISA kits with Catalog No. E-EL-R0842 obtained from Elabscience Biotechnology Inc. (Wuhan, Hubei, P.R.C., China).

#### Lipid profile and atherogenic indices

Triglycerides, TC and HDLc were estimated in the plasma, adipose and hypothalamus by standardized colorimetric methods using kits with Catalog No. BXC0271, BXC0261 and BXC0422A respectively obtained from Fortress Diagnostics Ltd. (Antrim, UK). Atherogenic indices were estimated as TG/HDLc and TC/HDLc in line with earlier description^51,53,76^.

#### Lipid peroxidation and antioxidant capacity

Malondialdehyde is a marker of lipid peroxidation and GSH reflect cellular antioxidant capacity of the cells. Malondialdehyde was determined from the plasma, adipose tissue and hypothalamus by standard non-enzymatic spectrophotometric method using assay kits from Randox Laboratory Ltd. (Co. Antrim, UK). This method involves the reaction of MDA in the sample with thiobarbituric acid (TBA) to generate MDA-TBA adduct, which was quantified spectrophotometrically, whereas GSH was determined using a non-enzymatic spectrophotometric method with assay kits obtained from Oxford Biomedical Research Inc. (Oxford, USA). Glutathione determination by spectrophotometric method was based on the oxidation of GSH in the sample by the sulfhydryl reagent 5,5′-dithio-bis (2-nitrobenzoic acid) (DTNB) to form the yellow derivative 5′-thio-2-nitrobenzoic acid (TNB), measured at 412 nm.

#### Glucoregulatory enzymes

Glucose-6-phosphate dehydrogenase activity was determined from the plasma, adipose tissue and hypothalamus using standard spectrophotometric method, whereas PDK4 level was determined from the plasma, visceral fat and hypothalamus using Rat ELISA kits obtained from Calbiotech Inc. (El Cajon, USA).

#### Nitric oxide concentration

Plasma and tissue NO were assayed spectrophotometrically by measuring the accumulation of its stable degradation products, nitrate and nitrite using kits from Oxford Biomedical Research Inc., (Oxford, UK). This kit employs the NADH-dependent enzyme nitrate reductase for conversion of nitrate to nitrite prior to the quantification of nitrite using Griess reagent—thus providing for accurate determination of total NO production.

#### Tumor necrosis factor-α and interleukin-6

Plasma and tissue concentration of TNF- α and IL-6 were determined by the quantitative standard sandwich rat ELISA technique using monoclonal antibody specific for these parameters using assay kits with Catalog No. E-EL-R0019 and Catalog No: E-EL-R0015 respectively, obtained from Elabscience Biotechnology Inc. (Wuhan, Hubei, P.R.C., China).

#### Histone deacetylase

The adipose and hypothalamic tissue levels of HDAC were determined using Rat ELISA kits with Catalog No. E0791Ra, obtained from Bioassay Technology Laboratory (Yangpu Dist. Shanghai 200,090, China), in compliance with the manufacturer’s procedure.

#### Peroxisome proliferator-activated receptor-γ

Plasma and tissue concentration of PPAR- **γ** was determined by the quantitative standard sandwich ELISA technique using monoclonal antibody specific for this parameter with rat assay kit (Catalog No. E-EL-R072) obtained from Elabscience Biotechnology Inc. (Wuhan, Hubei, P.R.C., China).

#### Pyruvate, lactate and γ-glutamyl transferase

Plasma and tissue concentration of pyruvate and lactate were determined by non-enzymatic standard colorimetric method using assay reagent kits obtained from Randox Laboratory Ltd. (Co. Antrim, UK) respectively. Whereas, plasma, adipose and hypothalamic GGT activity was measured by standardized enzymatic colorimetric method using assay kit obtained from Randox Laboratory Ltd. (Co. Antrim, UK).

#### Data analysis and statistics

The data were confirmed to be normally distributed using Shapiro–Wilk test and. All data were represented as means ± SD. Graphpad prism 9.2.0 was used to perform the statistical group analysis, and the mean values were compared using one-way ANOVA except for the oral glucose tolerance, where data were analyzed by repeated measures ANOVA. Then post hoc analysis was performed by Bonferroni’s test and statistically significant difference was determined at *p* < 0.05.

### Ethical statement

The study was conducted in accordance with the National Institutes of Health Guide for the Care and Use of Laboratory Animals and the protocol was approved by the Ethical Review Board of Afe Babalola University, Ado-Ekiti, Nigeria (ABUADERC/2020/06), and every effort was made to minimize both the number of animals used and their suffering.

## Supplementary Information

Supplementary Information.

## Data Availability

The data that support the findings of this study are available on request from the corresponding author.
