# Supplementary material for: Acetate rescues defective brain-adipose metabolic network in obese Wistar rats by modulation of peroxisome proliferator-activated receptor-γ
Source: Sci Rep. 2021 Sep 23;11:18967. doi: 10.1038/s41598-021-98605-5 (PMC8460633; doi:10.1038/s41598-021-98605-5)
Supplement: Supplementary file 1 — Supplementary Information. [file 41598_2021_98605_MOESM1_ESM.docx]

**Table 1: Initial and final body weight of experimental animals**

| **GROUPS** | **CTL** | **ACT** | **OBS** | **OBS+ACT** |
| --- | --- | --- | --- | --- |
| **Body weight (g)** |  |  |  |  |
| **Initial** | 172.71 ± 6.41 | 171.57 ± 7.28 | 171.00 ± 6.65 | 172.17 ± 5.75 |
| **Final** | 217.11 ± 8.71 | 202.61 ± 5.07 | 246.87 ± 7.30 | 211.19 ± 7.07 |
|  |  |  |  |  |
|  |  |  |  |  |

Data are expressed as mean ± S.D. n=6. Control (CTL), Acetate (ACT), Obesity (OBS).
